# Modeling and Analysis of Capacitive Relaxation Quenching in a Single Photon Avalanche Diode (SPAD) Applied to a CMOS Image Sensor

**DOI:** 10.3390/s20103007

**Published:** 2020-05-25

**Authors:** Akito Inoue, Toru Okino, Shinzo Koyama, Yutaka Hirose

**Affiliations:** Panasonic Corporation, 1 Kotari-yakemachi, Nagaokakyo City, Kyoto 617-8520, Japan; okino.toru@jp.panasonic.com (T.O.); koyama.shinzo@jp.panasonic.com (S.K.); hirose.yutaka@jp.panasonic.com (Y.H.)

**Keywords:** avalanche breakdown, avalanche photodiodes, CMOS image sensor (CIS), quenching, single photon avalanche diode (SPAD)

## Abstract

We present an analysis of carrier dynamics of the single-photon detection process, i.e., from Geiger mode pulse generation to its quenching, in a single-photon avalanche diode (SPAD). The device is modeled by a parallel circuit of a SPAD and a capacitance representing both space charge accumulation inside the SPAD and parasitic components. The carrier dynamics inside the SPAD is described by time-dependent bipolar-coupled continuity equations (BCE). Numerical solutions of BCE show that the entire process completes within a few hundreds of picoseconds. More importantly, we find that the total amount of charges stored on the series capacitance gives rise to a voltage swing of the internal bias of SPAD twice of the excess bias voltage with respect to the breakdown voltage. This, in turn, gives a design methodology to control precisely generated charges and enables one to use SPADs as conventional photodiodes (PDs) in a four transistor pixel of a complementary metal-oxide-semiconductor (CMOS) image sensor (CIS) with short exposure time and without carrier overflow. Such operation is demonstrated by experiments with a 6 µm size 400 × 400 pixels SPAD-based CIS designed with this methodology.

## 1. Introduction

Single-photon avalanche diodes (SPADs) are devices capable of detecting an individual photon by generating a large current pulse due to the Geiger mode (GM) avalanche multiplication of an electron-hole pair created on initial detection of a single photon. [1,2,3,4]. Rapid progress has been made in the development of SPAD-based complementary metal-oxide-semiconductor (CMOS) image sensors (CIS). Targeted applications include photon-counting imagers [5,6,7], scientific applications [8,9], and time-of-flight ranging sensors for autonomous driving systems [10,11,12]. In general, operation of a SPAD is comprised of three stages; (i) an idling stage; (ii) single-photon detection followed by a GM pulse generation; (iii) quenching the GM pulse. In the idling stage, a SPAD is biased with a voltage ($\left|V_{ex}\right|$) in “excess” of the breakdown voltage ($\left|V_{BD}\right|$). During the quenching process, generated charges accumulate on any capacitive component in parallel with the SPAD. This, in turn, brings the voltage across the terminals of the SPAD down to or below $\left|V_{BD}\right|$ where the quenching is completed [2,13,14]. Then, the accumulated charges are drained out through a series resistance in order to recover (recharge) the SPAD to the idling state. Conventional SPAD-based CISs have pixel circuits with many components for quenching and counting GM pulses resulting in relatively large pixel sizes and low fill factors [2,3,4,15]. However, if a SPAD-based CIS with a standard four-transistors (4T) pixel architecture could be applicable, then one could expect scaling advantages in system size and high resolution. [16] To this end, it is necessary to quench the triggered avalanche process under two conditions, i.e., (i) within a specified period of exposure and (ii) with a controlled amount of charges avoiding overflow. One attractive and probably the only solution, which satisfies the above two conditions simultaneously and what the authors use in this paper, called “capacitive quenching” is a kind of passive quenching (because it is not active quenching, so it has to be a kind of passive quenching, even though it is not resistive), similar to the resistive passive quenching (for example, [1,2,4]) where the resistance is set very high, thus making the quenching possible via the intrinsic diode capacitance and parasitic capacitances connected to the quench node. Then, a methodology to design the quenching period and the total amount of charges is required. The purpose of this paper is to present a quantitative design methodology of a SPAD-based CIS pixel incorporated into the 4T pixel circuit with the capacitive quenching capability. We present an analytical model of a SPAD, based on bipolar-coupled continuity equations (BCE) as used in references [2,13]. A model circuit is a parallel combination of a SPAD and a capacitor describing any parasitic and space charge components in series with a switch and a voltage source. We numerically solve the BCE of this model circuit from an idling stage to a complete quenching in the time domain. With a representative capacitance value, it is shown that generated charges can be stored on the capacitor within 200 ps order. More importantly, the total amount of the stored charges is found to be highly controllable as a function of the excess voltage, ~2$\left|V_{ex}\right|$, comprising the initial bias voltage $\left|V_{ex}\right|$ plus the reverse-excess voltage $\left|V_{re}\right|$ which is nearly equal to $\left|V_{ex}\right|$ pulled below the breakdown voltage. Thus, the amount of charges in the multiplication region is symmetric with respect to the breakdown voltage reaching the maximum when the internal bias returns exactly to $\left|V_{BD}\right|$. Thus, it is possible to design a potential profile of a SPAD to accommodate all the charges by setting up a barrier larger than 2$\left|V_{ex}\right|$. Experimentally, operation of a device, i.e., 400 × 400 6 µm pixels SPAD-based CIS, designed by using this method is demonstrated [17]. We note that the results obtained in the present work (both simulations and experiments) are different from the common sense of quenching of SPADs; “avalanche multiplication stops at the breakdown voltage.” This could be because; (1) this is the first time that the avalanche is analyzed with such equations, without assuming that avalanche stops at breakdown, (2) because in other works, SPAD was typically readout by oscilloscope or by any other component connected to it which introduces an additional stray capacitance, of few pF, and this can change the dynamics, (3) because instead in this case the SPAD is very small (6 µm) and with very small capacitance and no attached or very small parasitic capacitances, during avalanche build-up and quenching. Finally, the analysis is extended to a resistive quenching circuit and it is found that the driving force of the quenching is the same as that of the capacitive quenching, i.e., a field due to accumulated charges on the parallel capacitor.

## 2. Modeling of Capacitive Relaxation Quenching

As a representative model device, we consider a one-dimensional SPAD constituting a *p*-*i*-*n* structure biased into a Geiger mode regime, above the breakdown voltage, $\left|V_{BD}\right|$, and with the excess voltage of $\left|V_{ex}\right|$. Symbols describing physical quantities are listed in Table 1.

Important assumptions are; (1) the length of the *i*-region or the width of the depletion region is constant (=*W*); (2) carriers in the *i*-region transport with their saturation velocities, i.e., $v_{s,e}$ for electrons and $v_{s,h}$ for holes. As for condition (1), many SPADs are formed with a simple *p-n* junction, i.e., not the *p-i-n* structure. However, considering a large bias voltage and its small change after quenching, modulation of depletion width is estimated within ~5%, so that the assumption is reasonable. On the other hand, as for condition (2), since the field strength of the avalanche region is 3.5 × 10^5^ V/cm to 4.0 × 10^5^ V/cm and the material is silicon, constancy of saturation velocities is also reasonable [18].

With the definitions of field dependent ionization rates of electrons (=α(t)) and holes (=β(t)), time dependent numbers of electrons (=n(t)) and holes (=p(t)) in the entire *i*-region are described as follows.

(1) $$\frac{dn\left(t\right)}{dt}=\frac{n\left(t\right)}{\tau_{i,e}\left(t\right)}+\frac{p\left(t\right)}{\tau_{i,h}\left(t\right)}-\frac{n\left(t\right)}{\tau_{d,e}}$$

(2) $$\frac{dp\left(t\right)}{dt}=\frac{n\left(t\right)}{\tau_{i,e}\left(t\right)}+\frac{p\left(t\right)}{\tau_{i,h}\left(t\right)}-\frac{p\left(t\right)}{\tau_{d,h}}$$

where

(3) $$\frac{1}{\tau_{i,e}\left(t\right)}=\alpha \left(t\right)v_{s,e}$$

(4) $$\frac{1}{\tau_{i,h}\left(t\right)}=\beta \left(t\right)v_{s,h}$$

(5) $$\frac{1}{\tau_{d,e}\left(t\right)}=\frac{2v_{s,e}}{W}$$

(6) $$\frac{1}{\tau_{d,h}\left(t\right)}=\frac{2v_{s,h}}{W}$$

$\tau_{i,e}\left(t\right)$ and $\tau_{i,h}\left(t\right)$ are effective lifetimes or average inverse of ionization rates for electrons and holes. $\tau_{d,e}\left(t\right)$ and $\tau_{d,h}\left(t\right)$ are, respectively, the effective lifetime of electrons and that of holes in the *i*-region escaping out with the saturation velocities. The factor 2 derives from an assumption that carriers are generated in the middle of the *i*-region on the average. Equations (1) and (2) are represented by a matrix form as follows.

(7) $$\frac{d}{dt}N=TN$$

where

(8) $$N=\left(\begin{array}{c} n\left(t\right)\\ p\left(t\right) \end{array}\right)$$

and

(9) $$T=\left(\begin{array}{cc} \frac{1}{\tau_{i,e}}-\frac{1}{\tau_{d,e}} & \frac{1}{\tau_{i,h}}\\ \frac{1}{\tau_{i,e}} & \frac{1}{\tau_{i,h}}-\frac{1}{\tau_{d,h}} \end{array}\right)$$

are a carrier number vector and a transition matrix.

**Figure 1:**
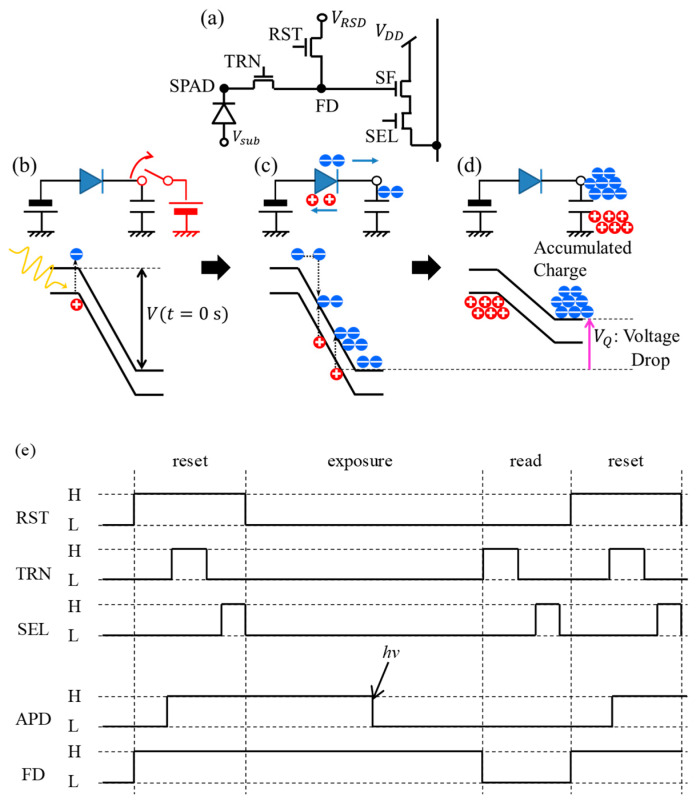
(**a**) Circuit diagram of a four transistors pixel circuit. Abbreviations TRN, RST, FD, SF, and SEL denote transfer transistor, reset transistor, floating diffusion, source follower transistor, and select transistor. (**b**–**d**) Simplified equivalent circuit models and band diagrams of single-photon avalanche diode (SPAD), (**b**) after the reset process, (**c**) during avalanche multiplication, and (**d**) after RQ. It is noted that the series capacitances in (**b**–**d**) are a summation of the diode capacitance and the stray components. (**e**) A typical timing chart of the pixel circuit. The notations, “H” and “L”, mean high voltage and low voltage is applied to gates of the transistors, respectively. The arrow denoted as *h*ν indicates an arrival of a photon during an exposure period resulting in voltage drop of the node SPAD.

We consider generation and quenching of a Geiger mode pulse assuming a SPAD applied to a CMOS image sensor (CIS) with a common four transistors circuit [17] as shown in Figure 1a. A fixed voltage is applied to the anode of the SPAD. By turning on the reset transistor (RST) and transfer transistor (TRN), the total SPAD bias is fixed and after turning off RST and TRN, the SPAD enters the exposure mode during which the cathode and the floating diffusion node (FD) are left floating. A simplified equivalent circuit and a band diagram of a SPAD after the reset are illustrated in Figure 1b. The series capacitance is actually, i.e., physically speaking, the capacitance intrinsic of the SPAD itself, i.e., of the depleted region. Upon a single photon detection, the SPAD generates a large current pulse by the avalanche effect (Figure 1c). With the increase of carrier accumulation in the series capacitor, the voltage of the floating node decreases, and the internal electric field of the SPAD is reduced. Eventually, the avalanche effect stops or is quenched as shown in Figure 1d.

The time dependence of the reverse voltage of the SPAD, |V(t)|, due to both the space charge and accumulated charge on the total capacitance of the SPAD cathode C is described as;

(10) $$\left|V\left(t\right)\right|=V_{0}-q\cdot \frac{n\left(t\right)+N_{c}\left(t\right)}{C}$$

where $\left|V_{0}\right|$ is an initial reverse bias of the SPAD (=|V(t=0 s)|), *q* is a unit electronic charge, and $N_{c}\left(t\right)$ is the number of charges accumulated on the capacitance satisfying the following relation

(11) $$\frac{dN_{c}\left(t\right)}{dt}=\frac{n\left(t\right)}{\tau_{d,e}}.$$

|V(t)| gives rise to reduction of the ionization field, (E(t)), in the *i*-region of the APD, and is related to the time-dependent ionization rates of electrons (α(E(t))) and of holes (β(E(t))) given according to the Chynoweth’s law [20].

(12a) $$\alpha \left(t\right)=\alpha_{0}\cdot \exp\left(-\frac{a}{E\left(t\right)}\right)$$

(12b) $$\beta \left(t\right)=\beta_{0}\cdot \exp\left(-\frac{b}{E\left(t\right)}\right)$$

where the values of $\alpha_{0}$,$\;\beta_{0}$, *a* and *b* due to Ref. [19] are listed in Table 1. Although more accurate expressions of impact ionization rates exist, for example references [21,22], our modeling at room temperature and electric field of 3.5 × 10^5^ V/cm to 4.0 × 10^5^ V/cm is covered by this choice of the ionization formula. *E*(*t*) is then given by

(13) $$E\left(t\right)=\frac{\left|V\left(t\right)\right|}{W}.$$

## 3. Results

### 3.1. Numerical Calculation

**Figure 2:**
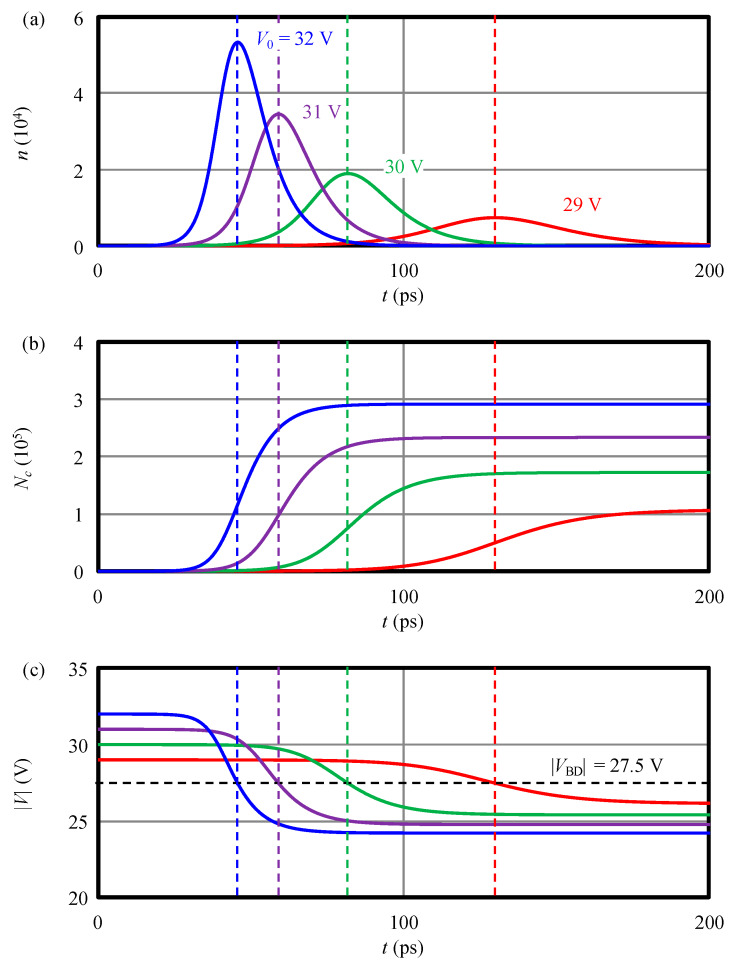
Calculated parameters in time domain. The time increment is 0.1 ps. (**a**) Time evolutions of the electron number in the *i*-region, (**b**) the number of accumulated electrons in the series capacitance, and (**c**) reverse bias of SPAD. The red, green, purple, and blue lines denote, respectively, the results with initial biases 29 V, 30 V, 31 V, and 32 V. A horizontal dashed line in (**c**) indicates $\left|V_{BD}\right|$ and vertical dashed lines show the times when d*n*/d*t* = 0 or $\left|V\left(t\right)\left|=\right|V_{BD}\right|$.

The calculated results with the device parameters in Table 1 and initial biases $\left|V_{0}\right|$ = 29–32 V are plotted in Figure 2. An initial condition is set as a situation in which an electron-hole pair is generated in the middle of the *i*-region assuming single-photon detection. At the beginning, the amount of charge in the *i*-region, *n*, increases with time as shown in Figure 2a. The carriers escaped from the depletion region are accumulated on the series capacitance (i.e., $N_{c}$ in Figure 2b) which, in turn, decreases the reverse voltage of SPAD as shown in Figure 2c. The time needed for this quenching process is short and is shown to be within 150 ps when $\left|V_{0}\right|$ is above 30 V. It should be noted that the time scale of the process is governed by the time-dependent time constants constituting the elements of matrix ***T*** of Equation (9), i.e., the rates of impact ionization and the escape velocities of carriers.

It is also interesting to note that, independent of $\left|V_{0}\right|$, when the reverse bias voltage drops to $\left|V_{BD}\right|$, the number of charge, *n*, reaches the maximum and correspondingly its time derivative equals zero as shown in Figure 2a (indicated by dashed vertical lines). This behavior reflects the nature of $\left|V_{BD}\right|$ as a point where the multiplication factor is infinite. After that point, *n* decreases and finally becomes zero.

The above analysis means the ionization continues even after the device voltage returns to the breakdown voltage because both electrons and holes still exist in the *i*-region and the impact ionization rates are still finite. Therefore, in order to completely quench the process, the reverse voltage must go below the breakdown voltage as $\left|V\left(t\to \infty \right)\right|=\left|V_{BD}\right|-\left|V_{re}\right|$, where $\left|V_{re}\right|$ expresses the voltage amount that should drop below the breakdown voltage. In order to accommodate all generated charges or, in other words, to prevent carrier overflow, the potential barrier of the storage capacitance $\left|V_{barrier}\right|$ must be higher than the voltage swing after the quenching, $\left|∆V_{Q}\right|=\left|V_{ex}\right|+\left|V_{re}\right|$. The condition is expressed as

(14) $$qN_{c}\left(t\to \infty \right)=C\cdot \left(\Delta V_{Q}\right)\leq C\cdot \left(\left|V_{barrier}\right|\right).$$

**Figure 3:**
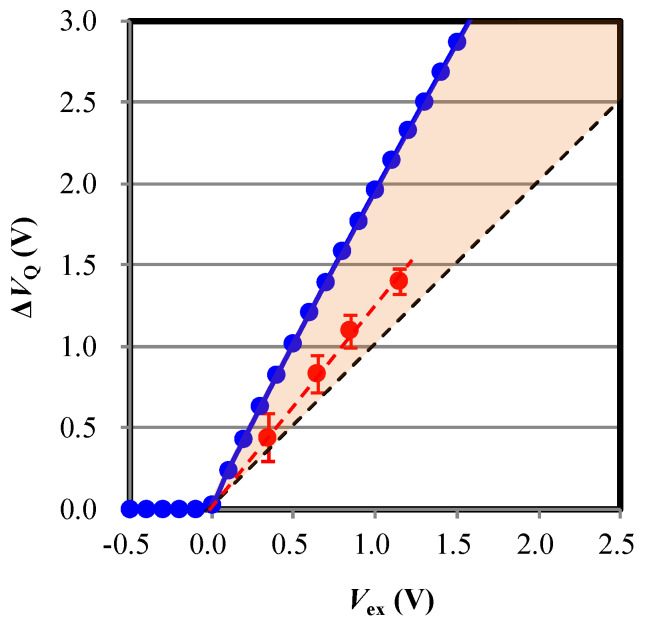
The voltage drop after the quenching ($=\Delta V_{Q}$) with respect to the initial bias ($=V_{0}$ ). The blue dots connected by line, the black dashed line, and the red circles connected by dashed line denote, respectively, the calculated results of $\Delta V_{Q}$, $\Delta V_{Q}=V_{ex}$, and the experimental result. A red hatched area is a region between calculated curves; $\Delta V_{Q}$ and $\Delta V_{Q}=V_{ex}$, where experimental results fall within. It is noted that the measured results of $\Delta V_{Q}$ are converted from the actually measured voltage of a sensing node or a floating diffusion (FD) by taking account of the capacitance values of a SPAD and FD.

We calculated $\left|∆V_{Q}\right|$ as a function of initial bias voltage and plotted as a blue line in Figure 3. As explained above, $\left|∆V_{Q}\right|$ is found to be larger than $\left|V_{ex}\right|$ (the black dashed line in Figure 3 indicating that $\left|∆V_{Q}\left|=\right|V_{ex}\right|$). The slope of the calculated $\left|∆V_{Q}\right|$ is about 2. This can be explained by the fact that, in Figure 2a, all the curves are nearly symmetric with respect to $\left|V_{BD}\right|$. This means that the same number of carriers are generated before and after the moment when the reverse bias reaches $\left|V_{BD}\right|$ resulting in the total voltage change of $“2”\times \left|V_{ex}\right|$.

It is noted that the above results are in contrast with a common quenching condition of $\left|∆V_{Q}\right|$~$\left|V_{ex}\right|$ [1,23,24]. There could be three possible reasons for such apparent difference; (1) use of the set of Equations (1)–(6) without assuming the cease of avalanching is scarcely done and rather unnoticed. In fact, other rare examples also with the introduction of a stochastic model of ionization at the breakdown voltage, they indeed found $\left|∆V_{Q}\right|>\left|V_{ex}\right|$ [14]. (2) because in other previous works, SPAD was typically readout by oscilloscope or by any other component connected to it which introduces an additional stray capacitance, of few pF, and this can change the dynamics. (3) because instead, in this case, the SPAD is very small (6 µm) and has very small capacitance and no attached or very small parasitic capacitances and resistance, during avalanche build-up and quenching. This is in high contrast to the assumption regarding the cathode voltage being nearly constant due to a large stray capacitance [24,25].

### 3.2. Experimental Results

**Figure 4:**
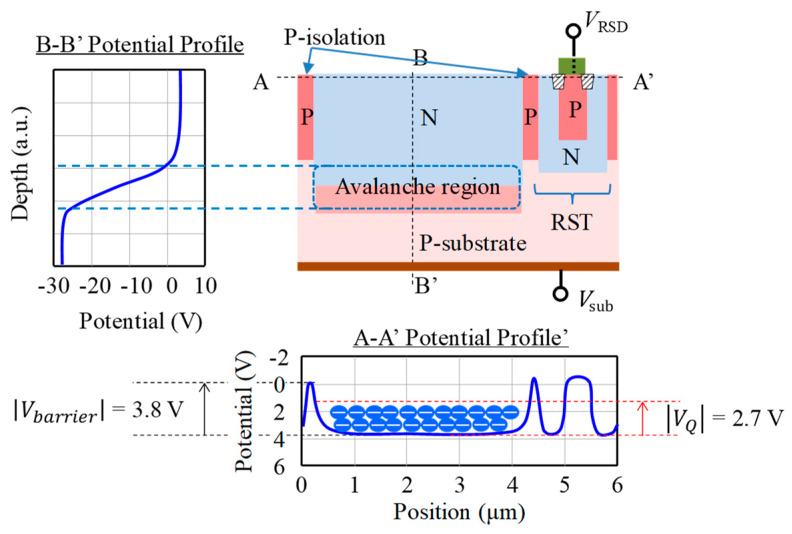
A cross sectional view of a SPAD with a vertical avalanche photodiode structure (VAPD) and the designed potential profiles in the horizontal (A-A’) and the vertical (B-B’) direction. The transistor shown on the cross section represents the reset transistor (RST), in Figure 1a.

To confirm the calculated results in the previous section, we measured the output voltage amplitude of the fabricated 6 µm 400 × 400 pixels SPAD-based CIS [17]. The specification of the device is summarized in Table 2. A cross-sectional view of the pixel and the simulated potential profiles in the horizontal and the vertical direction are illustrated in Figure 4. For this pixel, we set the barrier potential $\left|V_{barrier}\right|$ = 3.8 V to prevent overflow for the maximum $\left|V_{ex}\right|=1.5\;V$ and voltage swing of 2.7 V.

**Figure 5:**
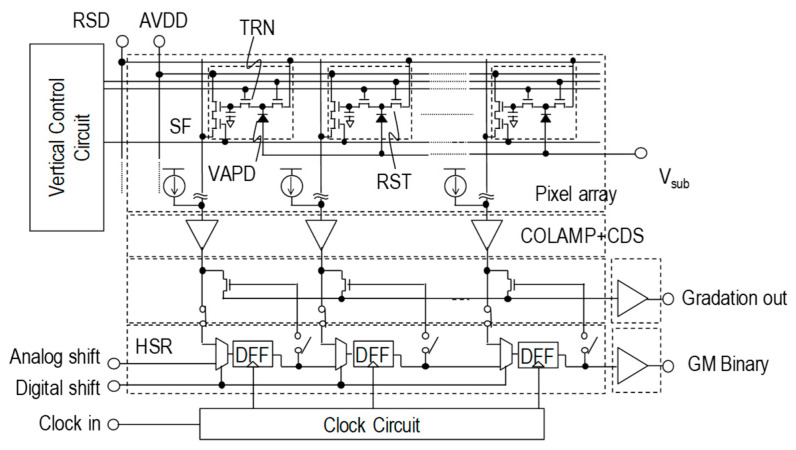
A block diagram of the developed CMOS image sensor (CIS).

A block diagram of the developed CIS is shown in Figure 5. The substrate voltage is externally controlled from 0 V for the normal imaging mode to −30 V for the Geiger mode. The shutter scheme is switchable between a rolling mode and a global mode. The readout path consists of the gradation image readout circuit for low gain modes and of the binary image readout circuit for the Geiger mode. The gradation image readout circuit comprises a column amplifier (COLAMP), correlated double sampling (CDS), and the horizontal shift registers (HSR). The Geiger mode binary signal is directly fed into the same HSR operated both as a binarizing circuit and as data-transferring flip-flops.

**Figure 6:**
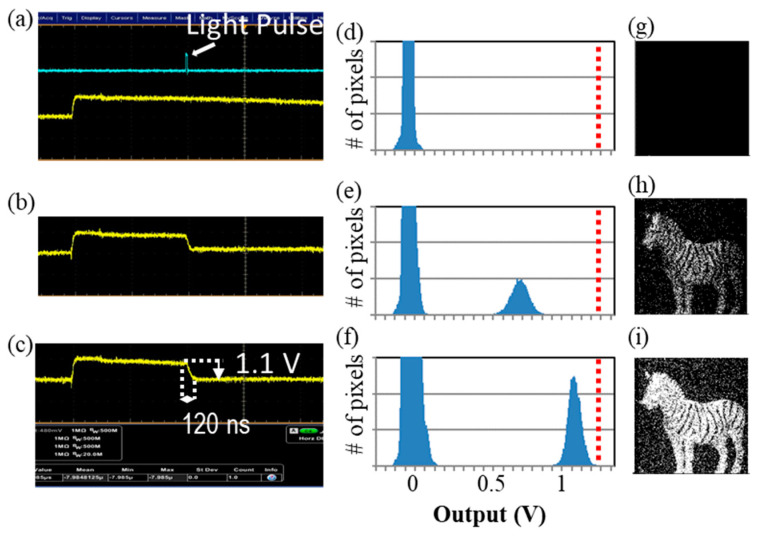
(**a**–**c**) Oscilloscope waveforms of output signals (Yellow: SF output, Blue: Light pulse) of pixels measured at $\left|V_{ex}\right|$ = (**a**) N.A. (non-avalanche region), (**b**) 0.7 V, (**c**) 1.2 V. (**d**–**f**) Histograms of output signal of pixels measured at $\left|V_{ex}\right|$ = (**d**) N.A. (non-avalanche region), (**e**) 0.7 V, (f) 1.2 V. A red line in the graph indicates SF saturation voltage (1.3 V). (**g**–**i**) Pictures of a zebra taken at $\left|V_{ex}\right|$ equals (**g**) N.A. (non-avalanche region), (**h**) 0.7 V, (**i**) 1.2 V. It is noted that the output voltage is slightly less than $\left|V_{ex}\right|$, e.g., 1.1 V with $\left|V_{ex}\right|$ = 1.2 V. Considering the gain of source follower (0.8), the input referred voltage swing is 1.4 V which is larger than $\left|V_{ex}\right|$.

Oscilloscope waveforms of the voltage amplitude at FD nodes under a low illumination condition (<1 nW/cm^2^) and 10 ns laser pulse (shown as a light blue line) with different bias conditions are plotted in Figure 6a–c. Histograms of the voltage amplitude at FD nodes with different bias conditions are plotted in Figure 6d–f. The object is illuminated by a near-infrared LED with 10 nW/cm^2^ intensity at the object plane. With normal mode conditions (Figure 6a), the signal amplitude is primarily due to random noise. In contrast, under the Geiger mode condition, $\left|V_{ex}\right|$ > 0.5 V, the output signal clearly shows binary feature exhibiting a large amount of carriers, i.e., a large amplitude of oscilloscope output and clearly separated peaks in the histogram. Since the highest signal level does not exceed the SF saturation level (1.3 V) plotted as a dashed red line in each plot, carrier multiplication is quenched with a finite signal level or without overflow. The pictures taken with the same bias conditions are shown in Figure 6g–i. The object can be seen only in Geiger mode pictures. It is noted that the output voltage is slightly less than $\left|V_{ex}\right|$, e.g., 1.1 V with $\left|V_{ex}\right|$ = 1.2 V. Considering the gain of source follower (0.8), the input-referred voltage swing should be 1.4 V about 1.2–1.3 times larger than $\left|V_{ex}\right|$. Thus, we not only observe that the output signal level dependence on $\left|V_{ex}\right|$, but also that output voltage swing indeed is estimated to be larger than $\left|V_{ex}\right|$, especially clearly observed in Figure 6c,f. It is also noted that the voltage drop period of 120 ns is longer than calculated 200 ps or less. This is due to a capacitance of the oscilloscope probe. Since during the exposure and quenching, the reset transistor is turned off and no current flows out of the FD node, this effect is not a problem for determining the output voltage value.

The measured $\left|\Delta V_{Q}\right|$, is plotted in Figure 3 as red circles in comparison to calculated results. The amplitude $\left|\Delta V_{Q}\right|$ of experiment exceeds $\left|V_{ex}\right|$ and $|\Delta V_{Q}\left|=1.3|V_{ex}\right|$. The maximum voltage swing is 1.3 V within the measurable range and the designed potential barrier is enough to avoid the overflow. The experimental result confirms the calculation that the voltage swing of quenching exceeds the excess bias. We discuss the reason that the experimental $\left|\Delta V_{Q}\right|$ is smaller than the calculation.

**Figure 7:**
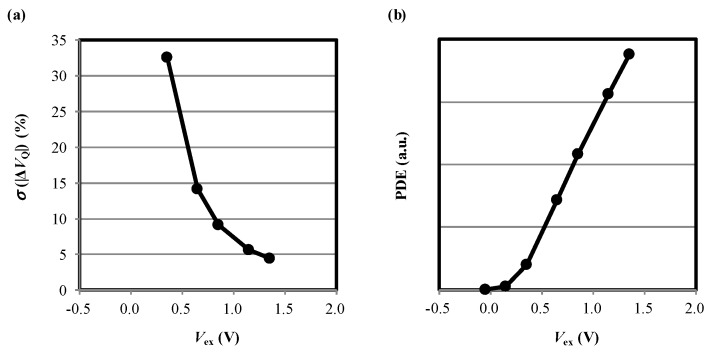
(**a**) The standard deviation of $\left|\Delta V_{Q}\right|$. (**b**) Photon detection efficient (PDE).

The standard deviations of $\left|\Delta V_{Q}\right|$ ($\sigma \left(\left|\Delta V_{Q}\right|\right)$) are analyzed by the histograms (Figure 6a–c) and are shown as error bars in Figure 3 and Figure 7a as a function of $\left|V_{ex}\right|$. Interestingly, $\sigma \left(\left|\Delta V_{Q}\right|\right)$ decreases with the increase of $\left|V_{ex}\right|$. We consider this result reflects a fact that the standard variation is affected more significantly with a situation where the carrier number is small or $\left|V_{ex}\right|$ is low. We also analyzed photon detection efficiency (PDE) of the SPAD as shown in Figure 7b (with arbitrary unit). PDE increases as $\left|V_{ex}\right|$ because of the rise of avalanche triggering probability [25].

## 4. Discussion

As mentioned above, avalanche multiplication doesn’t stop and the carrier number reaches the maximum value at the breakdown voltage. This is due to the character of the breakdown voltage where carrier generation and escape processes balance, i.e., equilibrium point. In this section, we discuss the breakdown voltage as an equilibrium point and derive formulas which universally hold at the breakdown voltage. We also discuss the difference between the experiment and the calculation. Furthermore, at the end of this section, we show the generality of the above model and consideration by analyzing BCE in a resistive quenching circuit.

### 4.1. Breakdown Voltage as an Equilibrium Point of the Dynamical System

When the bias voltage reaches the breakdown voltage $\left|V_{BD}\right|$, the time derivative of the carrier number is zero and the carrier number reaches the maximum value, i.e.,

(15) $$\frac{d}{dt}N=0.$$

The condition indicates balancing of impact ionization and the carrier drain processes. The bias voltage meeting this condition should be the breakdown voltage. Therefore, the state with the breakdown voltage is an equilibrium point which separates unstable (Geiger mode) and stable (linear mode) regimes.

We investigate the condition for Equation (16) to have a nontrivial solution. Equating the determinant of the matrix T (Equation (9)) to be zero, i.e., det |T| = 0, and by using Equations (3)–(6), we obtain the following simple expression

(16) $$\alpha \frac{W}{2}+\beta \frac{W}{2}=1.$$

It simply means that the breakdown occurs only when an electron and a hole cause one ionization event during their transport through the average distance of the *i*-region (= *W*/2). This is identical to the avalanche breakdown condition of the references [26,27] for a case where the ionization rates of electrons and holes are different. Correspondingly, the ratio of the electron and hole numbers in the *i*-region under this condition is calculated to be

(17) $$\frac{n}{p}=\frac{v_{s,h}}{v_{s,e}}=\frac{\tau_{d,e}}{\tau_{d,h}},$$

meaning that the ratio is inversely proportional to their velocities or proportional to the transport lifetimes. It is noted that the ratio does not depend on the ionization rates of electrons and holes.

### 4.2. Difference of $\left|\Delta V_{Q}\right|$ between Simulation and Experiment

**Figure 8:**
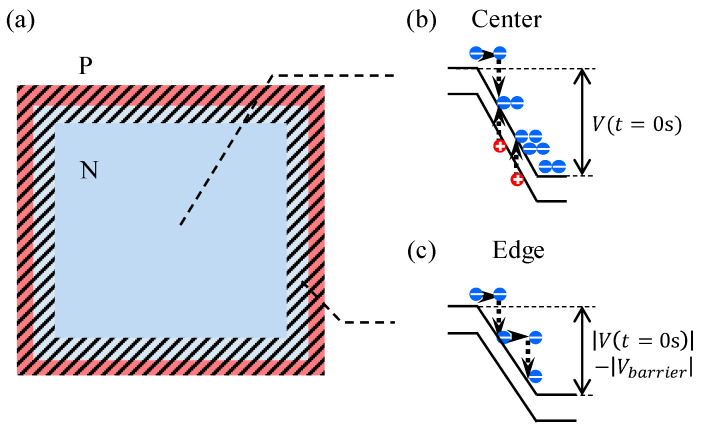
(**a**) A top view of a SPAD. The edge of the SPAD is hatched. (**b**,**c**) Simplified band diagrams at the center of the SPAD (**b**) and at the edge of the SPAD (**c**).

As shown in Section 3.2, the experimental $\left|∆V_{Q}\right|$ (the red circles in Figure 3) exceeds $\left|V_{ex}\right|$ but it does not reach the calculation result, $\left|\Delta V_{Q}\right|\approx 2\left|V_{ex}\right|$ (the blue line in Figure 3). This is probably due to spatial inhomogeneity of the field in the real device. Near toward the edge of a SPAD (hatched area in Figure 8a), the electric field is relatively low by the potential barrier, $\epsilon \left|V_{barrier}\right|$, where 0≤ϵ≤1. This is due to extension of depletion region from the surrounding isolation. Avalanche multiplication rarely occurs near the edge of the SPAD (Figure 8c), while $\left|\Delta V_{Q}\right|\approx 2\left|V_{ex}\right|$ consists around the center (Figure 8b). Because carriers diffuse perpendicular to the field direction during and/or after multiplication [28], the total value of $\left|∆V_{Q}\right|$ should fall between $\left|V_{ex}\right|$ and 2$\left|V_{ex}\right|$ as indicated by a red hatched area in Figure 3. Because of the experimental result, $\left|∆V_{Q}\right|≃1.3\left|V_{\mathrm{ex}}\right|$ (shown in Section 3.2), we consider the effective avalanche region is about 70% of the SPAD area reasonably agreeing with an effective fill factor (75%) estimated from a TCAD simulation.

### 4.3. The Mechanism of Resistive Quenching

**Figure 9:**
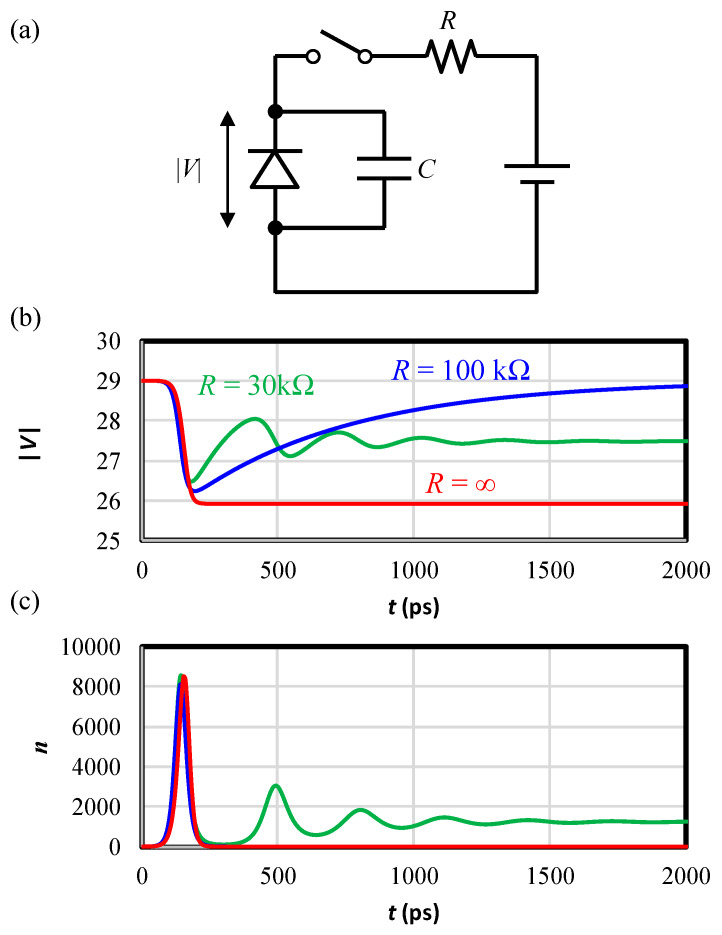
(**a**) A model circuit for a resistive quenching. (**b**) Calculated reverse biases for *R* = 100 kΩ (blue) *R* = 30 kΩ (green) *R* = ∞ (red). (**c**) Carrier numbers for *R* = 100 kΩ (blue) *R* = 30 kΩ (green) *R* = ∞ (red). Capacitance is 6fF for all conditions.

In order to comprehend the mechanism of quenching, we extend the BCE model to the resistive quenching circuit as schematically shown in Figure 9a. The amount of the accumulated carriers is rewritten from Equation (11) to

(18) $$\frac{dN_{c}}{dt}=\frac{n\left(t\right)}{\tau_{d,e}}-\frac{N_{c}}{RC}$$

where the second term of the right hand expresses current flowing through resistance, *R*.

The calculation was made on the same SPAD with $\left|V_{BD}\right|$ of 27.5 V. The calculated device terminal voltage and the number of carriers in the depletion region are plotted in Figure 9b,c, respectively, for three quenching resistances (*R* = 100 kΩ (blue curves) *R* = 30 kΩ (green curves) *R* = ∞ (red curves)). *R* = ∞ corresponds to the capacitive quenching. First to be noted is that, regardless of the values of R, from initial photo-carrier generation to quenching process gives nearly identical carrier evolution as indicated by the overlapped pulse curves below ~200 ps (Figure 9c). This is explained by discussions in Section 3.1; the overall time scale of the process is governed by the time constants given in matrix elements ***T*** of Equation (9). It is emphasized that the Geiger mode multiplication is completed or quenched by the completion of this carrier pulse completion. The driving force of the process is the bias field which is determined by the balance between the supply voltage and the field created by the carriers stored on the capacitance C. After the quenching, the bias recovers to the initial value with time constant RC. It is noted that the maximum swing of the terminal voltage with finite quenching resistance is reduced from that of the pure capacitive quenching, i.e., $2\left|V_{ex}\right|$ (Figure 9b). This is due to the fact that some of the generated carriers are lost through the resistance. The voltage swing of the capacitive quenching, i.e., $2\left|V_{ex}\right|$, is considered to be the worst case of voltage drop for a CIS pixel. Thus, for the purpose of preventing carrier overflow, the design criterion of the barrier potential (Equation (15)) is still applicable. When the resistance is R = 30 kΩ, the accumulated charge is drained to the voltage source before the completion of the ionization due to too fast carrier loss from the capacitance. And the terminal voltage goes back beyond $\left|V_{BD}\right|$ giving rise to another Geiger mode pulse. Thus, the carrier number oscillates with time. After the oscillation, steady current flows and the terminal voltage recovers only to $\left|V_{BD}\right|$. This corresponds to typically called “bad quenching” phenomenon and in line with the rule-of-thumb given in Ref. [24], that it is necessary to have at least 50 kΩ of quenching resistor for each volt of excess bias of the SPAD.

Therefore, in order completely to quench the Geiger mode multiplication and to recover to the initial state, recharge time constant, RC, should be longer than the time necessary for the quenching process (quenching time). In the present calculation where the quenching time is about 200 ps and the capacitance is 6 fF, a threshold resistance value is estimated to be about 33 kΩ in agreement with the results of Figure 9; successful quenching with 100 kΩ, and almost quenched but failed with 30 kΩ.

## 5. Conclusions

In conclusion, we have presented modeling and analysis of a Geiger mode pulse generation and of the capacitive relaxation quenching in a SPAD-based CIS. The carrier dynamics are modeled by BCEs. Numerical calculations of BCE show that the carrier number in a SPAD depletion region is maximum just at the breakdown voltage which reassures the role of the breakdown voltage as an equilibrium point. This, in turn, specified a necessary condition for successful quenching setting the final state being below $\left|V_{BD}\right|$ and the voltage swing is found to be nearly as twice large as $\left|V_{ex}\right|$ for a purely capacitive quenching. These analyses are essential to a design of SPAD-based CIS to avoid overflow. This design methodology is confirmed by experiments with the 400 × 400 6 µm pixels SPAD-based CIS. There is no overflow as confirmed by histograms of the images. This means that the signal level of images falls below the saturation level of the pixel output. We also show the driving force of the quenching is the same, i.e., reduction of the bias field due to carriers stored on the parallel capacitance, both in a resistive quenching circuit and in a capacitive quenching circuit demonstrating the generality of the presented modeling and analysis regardless of circuit types. 

## Figures and Tables

**Table 1:** Symbols and values of the physical constant quantities.

Meaning	Symbol	Value
Width of depletion region	*W*	0.80 µm
Saturation velocity of electron [19]	*v_s,e_*	1.02 × 10^7^ cm /s
Saturation velocity of hole [19]	*v_s,h_*	8.31 × 10^6^ cm/s
Coefficients of impact ionization ratio [18]	$\alpha_{0}$	3.80 × 10^6^ cm^−1^
	$\beta_{0}$	2.25 × 10^7^ cm^−1^
	*a*	1.75 × 10^6^ V/cm
	*b*	3.26 × 10^6^ V/cm

**Table 2:** Specifications of the fabricated SPAD-based CMOS image sensor (CIS).

CMOS Technology	65 nm 1P4M
Pixel Size	6 µm
Array size	400 × 400
Physical Signal	Photo-Charge
Quenching Type	Capacitive quenching
Fill Factor	70%
Operation Voltage	−3.3 V~−29 V
DCR(@RT)	100 cps
Frame Rate	60 fps
